# Comprehensive Surveys of Bean common mosaic virus and Bean common mosaic necrosis virus and Molecular Evidence for Occurrence of Other Phaseolus vulgaris Viruses in Tanzania

**DOI:** 10.1094/PDIS-01-18-0198-RE

**Published:** 2018-09-19

**Authors:** Beatrice Mwaipopo, Susan Nchimbi-Msolla, Paul J. R. Njau, Deogratius Mark, Deusdedith R. Mbanzibwa

**Affiliations:** Disease Control Unit, Mikocheni Agricultural Research Institute, Dar es Salaam, Tanzania;; Crop Science and Horticulture Department, Sokoine University of Agriculture, Chuo Kikuu, Morogoro, Tanzania;; † Disease Control Unit, Mikocheni Agricultural Research Institute, Dar es Salaam, Tanzania

## Abstract

Virus diseases are among the main biotic factors constraining common bean (Phaseolus vulgaris L.) production in Tanzania. Disease management requires information on types, distribution, incidence, and genetic variation of the causal viruses, which is currently limited. Thus, a countrywide comprehensive survey was conducted. Use of a next-generation sequencing technique enabled simultaneous detection of 15 viruses belonging to 11 genera. De novo assembly resulted in many contigs, including complete or nearly complete sequences of Bean common mosaic virus (BCMV), Bean common mosaic necrosis virus (BCMNV), and Southern bean mosaic virus (SBMV). Some viruses (for example, SBMV and Tomato leaf curl Uganda virus-related begomovirus) were detected for the first time in common bean in Tanzania. Visually assessed virus-like disease incidence ranged from 0 to 98% but reverse-transcription polymerase chain reaction-based incidence of BCMV and BCMNV (7,756 samples) was mostly less than 40%. The Sanger-based nucleotide sequences encoding coat proteins of BCMV and BCMNV isolates were 90.2 to 100% and 97.1 to 100% identical to each other, respectively. Phylogenetic analysis showed that BCMV isolates were more diverse than BCMNV isolates. The information generated in this study will contribute to the development of molecular diagnostic tools and strategies for management of virus diseases nationally and internationally.

Tanzania is the largest producer (>1,000,000 tons annually) of common bean (Phaseolus vulgaris L.) in sub-Saharan Africa (FAO 2014). Common bean serves as the main source of protein and starch for over 300 million people in East Africa and Latin America (Petry et al. 2015). In some parts of the African Great Lakes Region—for example, Kagera Region in Tanzania—a meal served without beans is considered an incomplete diet, which indicates the traditional values attached to it. The annual quantity of bean produced in Tanzania is associated with the large cultivated area rather than high yield per unit area (FAO 2014). In terms of yield, Tanzania is outperformed by several East African countries: Uganda, South Sudan, Madagascar, and Ethiopia (FAO 2014). The estimated yield of common bean for Tanzania is <1,000 kg/ha and the potential yield is 1,500 to 3,000 kg/ha (Hillocks et al. 2006; Nchimbi-Msolla 2013). Among other factors, the poor yields are attributed to fungal, bacterial, and viral diseases (Hillocks et al. 2006).

Many viruses are known to infect and cause diseases in common bean plants and can cause yield losses as high as 100% (Hagedorn and Inglis 1986). Bean common mosaic virus (BCMV) and Bean common mosaic necrosis virus (BCMNV) are considered the most important of these viruses (Grogan and Walker 1948). These viruses cause mosaic symptoms on common bean plants but BCMNV also causes systemic vascular necrosis, the disease also known as black root (Drijfhout 1978; Grogan and Walker 1948). At least five viruses—BCMV, BCMNV, Cowpea mild mottle virus (CPMMV; Carlavirus), Cucumber mosaic virus (CMV; Cucumovirus), and Cowpea aphid-borne mosaic virus (CABMV; Potyvirus)—have been detected either using enzyme-linked immunosorbent assays (ELISA) or differential cultivars in common bean plant samples collected from different parts of Tanzania (Davis and Hampton 1986; Mink and Keswani 1987; Mwaipopo et al. 2017; Njau and Lyimo 2000; Njau et al. 2006). Recently, using next-generation sequencing (NGS), two cryptic double-stranded RNA viruses belonging to the genus Alphaendornavirus—Phaseolus vulgaris alphaendornavirus 1 (PvEV-1) and Phaseolus vulgaris alphaendornavirus 2 (PvEV-2)— and CPMMV, a disease-causing virus, were detected in seed collected from farmers in three agricultural research zones of Tanzania (Nordenstedt et al. 2017). In neighboring countries, Peanut mottle virus (PeMoV) was detected in common bean samples collected from Zambia and Bean yellow mosaic virus from Kenya (Vetten and Allen 1991). Overall, this information suggests that common bean plants in East Africa are infected by many viruses.

There have been no comprehensive surveys of common bean virus diseases in Tanzania for the past 18 years. BCMNV and BCMV are perhaps the only viruses in Tanzania that have been surveyed adequately. Njau and Lyimo (2000) collected seed from farmers and research centers and showed that the highest incidences of BCMNV and BCMV were 36.6 and 12.4%, respectively. Vetten and Allen (1991) found that common bean samples collected from East Africa were mostly infected with BCMNV: in Tanzania, 23 of 60 samples were infected with BCMNV, 5 samples were infected with BCMV, and 2 samples were coinfected. In a different survey conducted in the 1990s, BCMNV incidence was low in southern but high in northern Tanzania; these geographical differences were associated with the vector population (Myers et al. 2000). Since then, distribution and incidence of common bean viruses have not been comprehensively studied.

## Funding

We acknowledge the Bill & Melinda Gates Foundation and the Government of Tanzania for financial support to Dr. Deusdedith Mbanzibwa through the Program for Emerging Agricultural Research Leaders (PEARL; contract ID OPP1112522).

*The e-Xtra logo stands for “electronic extra” and indicates that two supplementary figures and two supplementary tables are published online.

Common bean is grown in many geographically isolated parts of the country and, consequently, genetically distinct known and unknown viruses may occur. Molecular and biological information are required for determining genetic diversities of plant viruses, which then helps in developing diagnostic tools and predicting emergence of new strains or viruses. Furthermore, information on distribution and types of viruses that infect common bean is required for strategic breeding and deployment of planting material, as well as for guiding decision-making on imposing quarantine. In past research, ELISA was used to detect viruses in Tanzania (Mwaipopo et al. 2017; Njau and Lyimo 2000); unfortunately, a given antibody can only be used to detect a single virus or a group of very closely related viruses. Thus, many plant samples may test negative despite showing typical virus disease symptoms. NGS and Sanger sequencing techniques were employed to universally and simultaneously detect viruses in common bean plant samples collected during the first comprehensive countrywide survey. NGS is presently the most robust technique for detection of viruses and has unique power to universally detect viruses of all types, thus overcoming limitations of other plant pathogen detection methods (Boonham et al. 2014; Kehoe et al. 2014; Kreuze et al. 2009). Prior to this work, only one nucleotide sequence of a BCMNV isolate originating from Tanzania was available in GenBank (accession number HQ229995) (Larsen et al. 2011) and there were none for any other common bean viruses (Mwaipopo et al. 2017).

The objectives of this work were to (i) determine the distribution and incidence of virus-like disease symptoms, (ii) map the distribution of BCMV and BCMNV (the most commonly reported viruses from Tanzania), (3) universally detect viruses that infect common bean in Tanzania, and (4) generate molecular information for genetic diversity studies and development of diagnostic tools.

## Materials and Methods

**Fig. 1:**
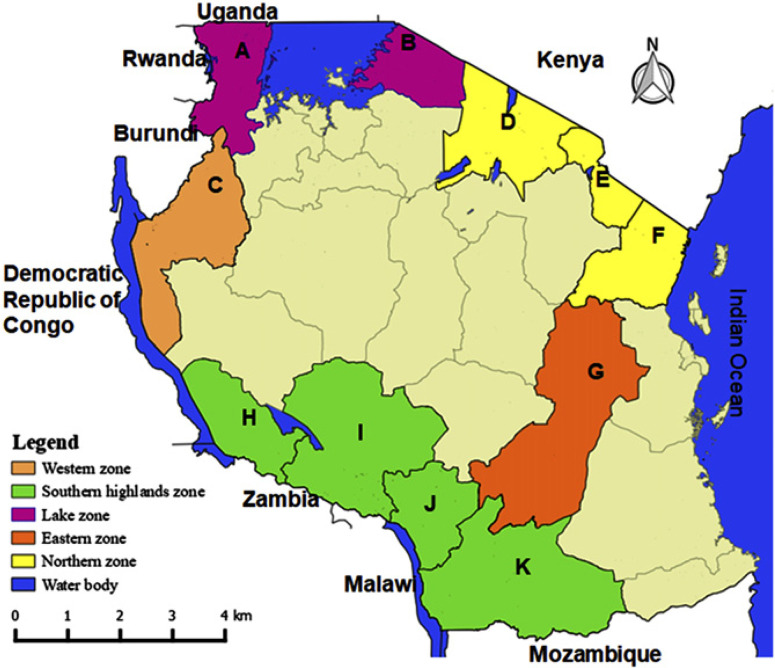
Locations surveyed for common bean viral diseases in Tanzania. Lake zone: **A**, Kagera Region (Ngara, Biharamulo, Muleba, Missenyi, and Karagwe Districts) and **B**, Mara Region (Tarime District). Western zone: **C**, Kigoma Region (Kasulu and Kibondo Districts). Northern zone: **D**, Arusha Region (Karatu and Arumeru Districts), **E**, Kilimanjaro Region (Siha and Hai Districts), and **F**, Tanga Region (Lushoto District). Eastern zone: **G**, Morogoro Region (Morogoro Rural, Mvomero, and Gairo Districts). Southern highlands zone: **H**, Rukwa Region (Nkasi District), **I**, Mbeya and Songwe Regions (Mbozi, Mbeya Rural Districts), J, Njombe Region (Njombe Rural and Wanging’ombe Districts), and K, Ruvuma Region (Namtumbo and Mbinga Districts).

Survey areas and field selection. Surveys were carried out in 23 administrative districts (herein called districts) in five national agricultural research zones (herein called agricultural research zones) in Tanzania. These 23 districts are found in 12 administrative regions, which border eight countries (Fig. 1). In each district, 4 to 15 common bean fields located near main or feeder roads were randomly selected for sample collection and visual assessment of viruslike disease incidence. The distance between sampled fields was a few hundred meters to 10 kilometers or more, depending on the availability of common bean fields. A geographical positioning system (GPS 72H; Garmin, Taiwan) was used to record coordinates for all surveyed fields. The maps of surveyed areas were then generated from the coordinates using Quantum GIS v. 2.6.

Sample collection and visual assessment of virus-like disease incidence. In each selected common bean field, virus disease incidence was visually assessed by observations on 50 randomly selected plants. Five points were randomly chosen in each field and observations of virus-like disease symptoms were made on 10 plants at each point. The plants were scored as either diseased or not, and symptoms were recorded in a field disease survey sheet. The incidence for virus-like disease symptoms was then computed as the percentage of plants exhibiting symptoms (i.e., by dividing the number of plants with symptoms per field by the total number of plants on which observations were made). Overall disease symptom incidence for each district was calculated by dividing the total number of plants with symptoms by the total number of plants on which observations were made in that particular district.

Reverse-transcription polymerase chain reaction-based determination of BCMNV and BCMV incidence. From each surveyed field, 30 leaf samples (except in the western zone, where only 10 to 13 samples were collected per field) were randomly collected from 30 different plants, randomly selected from among the 50 common bean plants on which symptoms were observed. The collected leaf samples were placed in sampling bags containing silica gel. Another sample from the same plants was placed between two flip-chart paper sheets and pressed using a plant press and stored at room temperature until use. In total, 7,756 common bean leaf samples were collected from 279 fields and brought to the laboratory at the Mikocheni Agricultural Research Institute (MARI). RNA was extracted from each sample as explained below. Then, reverse-transcription polymerase chain reaction (RT-PCR) was performed. RT-PCR-based incidence was calculated as the percentage of plants singly infected with BCMNV and BCMV. All plants infected with each virus were summed up to calculate incidence for each virus in each district. Samples infected with both viruses were recorded after determining individually infected samples. In each district, prevalence for BCMV and BCMNV was recorded as the percentage of fields with infected plants.

RNA extraction. RNA was extracted from either silica gel desiccated or plant-press pressed dry common bean leaf samples using a modified cetyltrimethylammonium bromide (CTAB) method. The CTAB buffers contained 2% CTAB, 100 mM Tris-HCl, 20 mM EDTA, 2.5 M NaCl, freshly prepared 1% sodium sulfite, 2% polyvinylpyrrolidone, and 2.5% 2-mercaptoethanol in nuclease-free water. Other procedures were as reported recently for RNA extracted at MARI (Nordenstedt et al. 2017). The integrity of RNA in the samples was assessed visually by agarose gel electrophoresis after staining the gel with ethidium bromide. RNA concentration and purity were determined with a Nanodrop 2000c UV-vis spectrophotometer (Thermo Scientific, Wilmington, DE, USA).

Complementary DNA synthesis. The first-strand complementary DNA (cDNA) synthesis was done using Moloney Murine Leukemia Virus reverse transcription (number M0253; New England Biolabs [NEB], Ipswich, MA, USA) following a modified manufacturer’s protocol. The first mix contained 1 mg of RNA, 1 ml of 100 mM oligo (dT)18 or random hexamer (Bioneer, Seoul, South Korea), 1 ml of dNTP, and diethyl pyrocarbonate-treated water to a volume of 10 ml. The mixture was heated at 65 °C for 5 min and spun down after chilling on ice for 2 min. The second master mix was prepared according to NEB’s standard protocol. Total volume for cDNA synthesis reaction was 20 ml. Random hexamer and oligo dT18-21 primed reactions were incubated at 37 and 42°C, respectively, for 90 min, and enzymes were inactivated at 65 °C for 20 min. For determination of virus incidence, equal amounts of RNA extracted from five plants collected from the same field were pooled prior to cDNA synthesis. The pools that tested positive were marked and previous plant RNA samples were retrieved and tested individually by RT-PCR to identify the infected plant samples.

PCR. To determine incidences of BCMV and BCMNV, PCR were run using different pairs of primers (Table 1). These primers were initially designed using sequences of BCMNV and BCMV retrieved from GenBank and later using some sequences obtained in this study. A high-fidelity Phusion DNA polymerase (number M0530S; NEB) was used when PCR was run to generate PCR products for Sanger sequencing and AccuPower PCR PreMix (K-2036; Bioneer) was used for routine detection of BCMV and BCMNV in samples. For Phusion DNA polymerase, the 50 ml PCR contained 10 ml of 5x Phusion GC buffer, 1 ml of dNTP (10 mM), 2 ml of each forward and reverse primer (10 mM), 0.5 ml of Phusion DNA polymerase (2 U/|xl), and 5 ml of cDNA template. The primer pairs used to amplify BCMV and BCMNV for sequencing were BCMVFcpF1/BCM-NVcommonR or BCMVFcpF1/BCMVFcpR1 and BCMNVFcpF2/BCM-NVcommonR or BCMNVF1/BCMNVR1, respectively (Table 1). The PCR program was the same for the four pairs of primers; initial denaturation was at 98°C for 30 s, followed by 32 cycles at 98°C for 5 s, 57°C for 20 s, and 72°C for 30 s. Final extension was at 72°C for 10 min. Two primer pairs (533-340F1/ 533-340R1 and 533-139F1/533-139R) were designed to amplify BCMV in order to compare sequences obtained by NGS and Sanger sequencing data for isolate TZ:Mor533:2017. These primers annealed at 60°C. The primer pair ToLCUV-F1/ToLCUV-R1 was used to detect a begomo-like virus in common bean samples (Table 1). The PCR for this primer pair contained forward and reverse primers, dNTP, and Phusion DNA polymerase at the concentrations shown for other primer pairs when Phusion DNA polymerase was used. The DNA template used was 25 ng per PCR. The initial denaturing was done at 98 °C for 30 s followed by 35 cycles of amplification with denaturation at 98°C for 5 s, annealing at 56°C for 30 s, and extension at 72°C for 30 s. The final extension was done at 72°C for 10 min.

**Table 1:** Primers designed and used in this study

Primer name (in pairs)	Primer sequences 59–39 direction	Product size (bp)	Targeted region^a^	Virus^b^
BCMVFcpF1	GCGGAGAATCTGTGCACCTACA	…	…	…
BCM-NVcommonR	GTCCCKTGCAGTGTGCCT	839	CP	BCMV
BCMVFcpF1	GCGGAGAATCTGTGCACCTACA	…	…	…
BCMVFcpR1	ATTGCAATGGTTCTTCCGGC	1075	CP and 3¢UTR	BCMV
BCMNVFcpF2	GCTGGGGCCGATGAGAG	…	…	…
BCM-NVcommonR	GTCCCKTGCAGTGTGCCT	711	CP	BCMNV
BCMNVF1	CAAAGGCCCAGCGGATAAA	…	…	…
BCMNVR1	GGTGGTATAACCACACTGGAATTG	823	CP and 3¢UTR	BCMNV
BCMV1F	GTAGCACAGATGAAGGCAGCA	…	…	…
BCMV1R	GGTTCTTCCGGCTTACTCATA	339	CP and 3¢UTR	BCMV
533-340F1	GCTGGAACAGCTCACCAA	…	…	…
533-340R1	CCTTTGATTCTCTCTGCCTTT	668	P3	BCMV; TZ:Mor533:2017
533-139F1	GTCAAGCAAGCAAAGAGTGC	…	…	…
533-139R1	TGTGTAATCCCTCAAATACCGC	546	CI	BCMV; TZ:Mor533:2017
ToLCUV-F1	GTGAATCCCCAATTCCTTCCTC	…	…	…
ToLCUV-R1	TCCCACTATCTTCCTCTGCAA	434	C2 and C3	ToLCUV

a CP, UTR, P3, and CI indicate potyviral coat protein, untranslated region, third protein, and cytoplasmic inclusion, respectively, while C2 and C3 refer to transcriptional activation and replication enhancement proteins of Tomato leaf curl Uganda virus (ToLCUV)-related begomovirus detected in this study.

b TZ:Mor533:2017 is an isolate of *Bean common mosaic virus* (BCMV). BCMNV = *Bean common mosaic necrosis virus*.

AccuPower PCR PreMix was used in routine detection of BCMNV and BCMV and determination of incidence using primer pairs BCMNVF1/BCMNVR1 and BCMV1F/BCMV1R, respectively. To a 0.2 ml tube containing AccuPower PCR PreMix, 15 ml of diethyl pyrocarbonate-treated water, 1 ml of each of the forward and reverse primers (10 mM), and 3 ml of cDNA template were added to make a total volume of 20 ml. For both pairs of primers, the PCR program was as follows: initial denaturation was for 2 min at 94°C; followed by 35 cycles at 94°C for 25 s, 50°C for 30 s, and 72°C for 1 min (primerpair BCMNVF1/BCMNVR1) or 30 s (primer pair BCMV1F/BCMV1R); and final extension at 72°C for 10 min. The PCR products were run in a 1% agarose gel stained with ethidium bromide. Gel images were captured using a Benchtop UV Transilluminator (UVP; Upland, CA, USA) under UV light. Gel images were used to score for infection status, and BCMV and BCMNV incidences were calculated as percentages of samples infected with each virus, with the sampling domain being first a field and then a district (Sseruwagi et al. 2004).

Sequencing of PCR products. PCR products were sequenced at three different facilities: Haartman Institute (Finland), Mbeya Zonal Referral Hospital Laboratory (Tanzania), and Bioneer. PCR products were purified using PCR purification kits (Bioneer) or treated with exonuclease I and calf intestinal alkaline phosphatase (NEB) following the enzyme manufacturers’ instructions, then sequenced on both strands.

Sequencing of small RNA and data analysis. Equal amounts (7 mg) of total RNA extracted from 30 plants from each of the five zones were separately pooled to make six (two samples for the Lake zone) zonal pooled RNA samples: HXH-1 (southern highlands zone), HXH-2 (eastern zone), HXH-3 (northern zone), HXH-6 (also contained RNA from cassava plants), and HXH-7 (Lake zone), and HXH-15 (western zone). Moreover, there were two nonpooled RNA samples (HXH-4 and HXH-5), and the HXH-4 sample also contained RNA extracted from cassava, a subject for another study. The 30 leaf samples from which RNA was extracted for NGS were selected based on severity or uniqueness of disease symptoms observed on common bean plants in fields but, for each pool, it was ensured that at least a sixth of the samples were from asymptomatic plants. For sequencing, total RNA was shipped on dry ice to Fasteris SA in Switzerland. Then, the small RNA were isolated (acrylamide gel size selection) and cDNA libraries were prepared and sequenced using Illumina HiSeq 2500 (Illumina Inc., San Diego, CA, USA) as described previously (Mbanzibwa et al. 2014; Nordenstedt et al. 2017). Analyses of NGS data were done using the VirusDetect program (v.1.6 and v.1.7) (Zheng et al. 2017) on a supercomputer (https://www.csc.fi; Finland) accessed between January 2016 and October 2017. The files received from Fasteris SA were unzipped using the command “tar-vxf filename”. Then, all reads of sizes not within 21 to 24 nucleotides (nt) were deleted. The remaining reads were analyzed using two approaches: de novo assembly was first done on each read size separately and, later, the inserts for these four sizes were combined (for simplicity herein called “combined inserts”) using the command “cat *.fastq > filename.fastq’ to obtain one fastq file and assembled using the command “virus_detect.pl *.fastq” (for offline analysis, the command was “perl virus_detect. pl filename”). Offline analysis on desktop or laptop computers (random access memory of 8 GB; installed with virtual Linux machine) using VirusDetect v.1.6 was possible for “not combined” reads but failed for some combined inserts (i.e., inserts of sizes 21 to 24 nt as a single fastq file). The contigs obtained were inspected for open reading frames using the Expasy-translate tool (https://www.expasy. org/). To obtain and manually edit longer nucleotide sequences, contigs obtained by analyzing inserts of sizes 21 or 22 nt were aligned against identical contigs obtained through analysis of combined inserts. For the nonpooled sample HXH-4 (isolate TZ: Mor533:2015), the contigs that mapped to the same reference sequence or to too-closely related virus sequences were assembled using the SeqMan program (v.5.03; DNASTAR, Madison, WI, USA) and cross-checked using Sanger sequencing.

NGS raw data received from Fasteris SA was submitted at Zenodo and assigned DOI 10.5281/zenodo.841170. Sanger and NGS sequences were submitted to GenBank and inserts of sizes 21 to 24 nt were submitted to the European Nucleotides Archive. Accessions are shown in Supplementary Table S1.

**Sequence analysis.** Phylogenetic analysis was achieved using MEGA7 software (Kumar et al. 2016). The coat protein (CP)-encoding nucleotide sequences of BCMV and BCMNV were first aligned using the MUSCLE program (Edgar 2004) and trimmed to equal size (620 nt). Then, the evolutionary history was inferred using the maximum-likelihood method based on the Tamura-Nei model (Tamura and Nei 1993). The tree with the highest log-likelihood was used. Initial trees for the heuristic search were obtained automatically by applying Neighbor-Join and BioNJ algorithms to a matrix of pairwise distances estimated using the Maximum Composite Likelihood approach, and then selecting the topology with superior log-likelihood value.

Nucleotide and amino acid sequence identities were determined using the BioEdit Sequence Alignment Editor (Hall 1999). Translation of nucleotide sequences into protein sequences was achieved using the “translate” option in MEGA7. Putative cleavage sites in potyviral sequences were predicted as described by Adams et al. (2005); for other viruses, comparisons were made to previously annotated sequences.

## Results

**Fig. 2:**
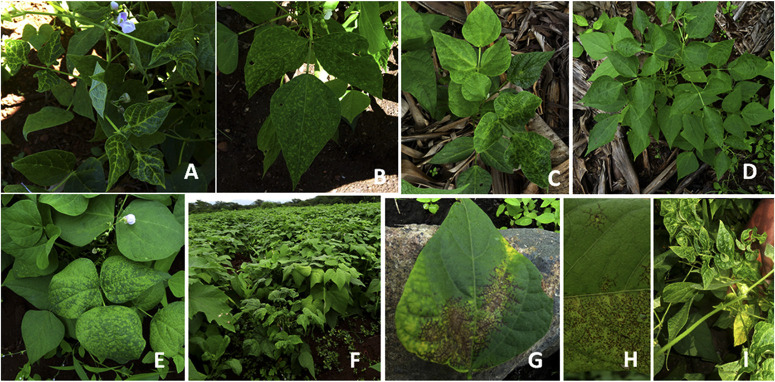
Some symptoms observed on common bean plants in fields during 2015 to 2017. **A**, Vein clearing in plants infected with Southern bean mosaic virus (Kasulu District in the western zone). **B**, Yellow mottling (Missenyi District in Lake zone). **C** and **D,** Mosaic and leaf curl (Missenyi District in Lake zone). **E**, mosaic (Karatu District in northern zone). **F**, Mosaic and stunted growth (northern zone). **G** and **H**, leaf necrosis (Kilimanjaro Region in northern zone). **I**, Mosaic and severe leaf deformation (Mvomero District in eastern zone).

**Virus disease symptoms on plants in fields.** Virus and virus-like disease symptoms were observed on common bean plants in fields across the country. The symptoms included vein banding (green and yellow); vein clearing; leaf distortion, puckering, and rugosity; mosaic; upward and downward leaf curl; stunted growth; purpling; mild to conspicuous yellow spots or patches; mottling; and necrosis on leaves (Fig. 2). These disease symptoms were observed in the five agricultural research zones but severe necrosis on leaves was most common in Kilimanjaro Region of the northern zone. Vein clearing was most common on leaves in the Kigoma Region of the western zone.

**Visual incidence of virus and virus-like disease symptoms.** The incidence of virus and virus-like disease symptoms was assessed visually on 50 plants per common bean field. Incidence was in the range of 0 to 86,6 to 76, 0 to 94,4 to 98, and 0 to 80% in the southern highlands, eastern, northern, Lake, and western zones, respectively (Table 2). When incidence was calculated at a district level (i.e., dividing total number of diseased plants by total number of examined plants in each district), the range was 11.0 to 43.1%: the extremes in this range for incidence were in Tarime (Lake zone) and Mvomero (eastern zone) districts, respectively. There were no plants with virus disease symptoms in 46.7% of assessed fields in the western zone (data not shown). In the northern zone, there was only one field with 0% virus disease symptoms incidence and, in the southern highlands zone, there were five fields with no observable virus disease symptoms (6.2%). In the eastern and Lake zones, virus disease symptoms were observed in all common bean fields.

**Table 2:** Visual and reverse-transcription polymerase chain reaction (RT-PCR) based incidence and prevalence of Bean common mosaic necrosis virus (BCMNV) and Bean common mosaic virus (BCMV) in common bean plants in different agricultural zones of Tanzania

Zone^a^	District^b^	N^c^	RT-PCR-based disease incidence range at field level (%)		RT-PCR-based		Incidence (%)^d^
			BCMNV	BCMV	BCMNV	BCMV	
SHZ	Nkasi (H)	450	0-10.0	0-13.3	6.7	13.3	0-86
	Mbozi (I)	420	0-3.3	0	7.1	0	10-60
	Mbeya Rural (I)	420	0-10.0	0-6.7	28.6	14.3	0-80
	Njombe (J)	330	0	0-26.7	0	36.4	0-30
	Wanging’ombe (J)	150	0	0	0	0	6-48
	Mbinga (K)	450	0	0	0	0	2-42
	Namtumbo (K)	300	0-3.3	0	10.0	0	6-44
EZ	Gairo (G)	450	0-76.7	0-26.7	13.3	13.3	6-54
	Mvomero (G)	450	0-40.0	0-36.7	33.3	33.3	12-76
	Morogoro R. (G)	120	0-3.3	0	50.0e	0	18-24
NZ	Karatu (D)	450	0-16.7	0-20.0	20.0	46.7	2-86
	Arumeru (D)	420	0-3.3	0-26.7	7.1	28.6	6-94
	Siha (E)	240	0-23.3	0-33.3	25.0	50.0	2-34
	Hai (E)	210	0-36.7	0-20.0	28.6	28.6	0-52
	Lushoto (F)	420	0-30.0	0-36.7	14.3	21.4	2-56
LZ	Ngara (A)	240	0	0-3.3	0	12.5	12-36
	Karagwe (A)	450	0	0-10.0	0	26.7	4-54
	Missenyi (A)	450	0-16.7	0-16.7	13.3	13.3	8-98
	Muleba (A)	450	0	0	0	0	18-64
	Biharamulo (A)	150	0	0-10.0	0	20.0	4-38
	Tarime (B)	420	0-3.3	0-36.7	14.3	7.1	4-22
WZ	Kasulu (C)	163	0	0	0	0	0-80
	Kibondo (C)	153	0-3.3	0	0	0	0-60

a SHZ, EZ, NZ, LZ, and WZ indicate agricultural research zones in Tanzania: southern highlands, eastern, northern, Lake, and western zones, respectively. Surveys in SHZ, EZ, NZ, and LZ were carried out in 2015, whereas the WZ survey was conducted in 2017.

b Letters in parentheses represent locations: A, Kagera Region; B, Mara Region; C, Kigoma Region; D, Arusha Region; E, Kilimanjaro Region; F, Tanga Region; G, Morogoro Region; H, Rukwa Region; I, Mbeya and Songwe Regions; J, Njombe Region; and K, Ruvuma Region.

c Indicates the number of common bean samples collected from each district and on which RT-PCR was performed.

d Visually assessed incidence of virus-like disease symptoms in 50 plants per field.

e Only four common bean fields were surveyed in Morogoro Rural District.

**RT-PCR-based incidence and prevalence of BCMNV and BCMV.** At field level, RT-PCR of 7,756 common bean samples from the five agricultural research zones revealed virus incidence in the range of 0 to 76.7% for BCMNV and 0 to 36.7% for BCMV (Table 2; Supplementary Fig. S1). The field with the highest incidence (76.7%) of BCMNV was in Gairo District in the eastern zone. Three fields had the highest BCMV incidence (36.7%), in three different districts: Mvomero, Lushoto, and Tarime in the eastern, northern, and Lake zones, respectively. Only 1 sample out of 316 leaf samples collected from Kigoma Region—on the border with Burundi—was infected with BCMNV, and none were infected with BCMV.

Of the 248 fields surveyed in the eastern, northern, southern highlands, and Lake zones, 218 and 204 fields had no plants infected by BCMNV and BCMV, respectively. In each of the districts covered under this study, there was at least one field in which all common bean plant samples collected were free of BCMNV and BCMV infections (Table 2). The infected plants were mainly found concentrated in a few areas in fields.

Coinfections of BCMNV and BCMNV were rare. Most plants with coinfections were from Siha District in the northern zone, in which four common bean plants from the same field were coinfected. In the same zone but in Arumeru District, only one plant was coinfected. In the eastern zone, only two plants from two different common bean fields were coinfected. No BCMV and BCMNV coinfections were detected for samples from the western, Lake, and southern highlands zones.

**Viruses detected by NGS.** RNA extracted from common bean samples was pooled separately for each zone and sent for sequencing at Fasteris SA. Briefly, 30 RNA samples from 30 different symptomatic and nonsymptomatic plants were pooled to make a sample for each zone (except that two pools were made for the Lake zone). In total, RNA from 180 individual symptomatic and asymptomatic plants was sequenced by targeting virus-derived small RNA of sizes to 24 nt, which are generated along with genomic small RNA during plant defense (Baulcombe 2004; Kreuze et al. 2009; Mlotshwa et al. 2008). Deep sequencing of the small RNA resulted in 5.87, 6.67, 5.28, 11.66, 7.99, and 28.22 million reads of sizes 21 to 24 nt for samples HXH-1, HXH-2, HXH-3, HXH-6, HXH-7, and HXH-15, respectively (Table 3). Of these, 80,846 (HXH-7) to 3,793,494 (HXH-15) reads were aligned to the reference virus sequence database.

**Table 3:** Viruses detected by deep sequencing of virus-derived small RNA from common bean samples collected from five different agricultural research zones in Tanzania

Zone^a^	RNA pool sample	Total reads (21–24 nucleotides)	Reads aligned to reference	Viruses detected (reference sequences and percent coverage are shown in parentheses)^b^
SHZ	HXH-1	5,869,348	370,969	BCMNV; +ssRNA; Potyvirus (AY864314; 95.1)
	…	…	…	PeMoV; +ssRNA; Potyvirus (AF023848; 98.6)
	…	…	…	SBMV; +ssRNA; Sobemovirus (DQ875594; 99.9)
	…	…	…	PvEV-1; dsRNA; Alphaendornavirus (KT456287; 95.0)
	…	…	…	PvEV-2; dsRNA; Alphaendornavirus (AB719398; 96.4)
EZ	HXH-2	6,674,109	264,061	BCMNV; +ssRNA; Potyvirus (AY864314; 93.4)
	…	…	…	BCMV; +ssRNA; Potyvirus (KT175569; 100)
	…	…	…	PvEV-1; dsRNA; Alphaendornavirus (KT456287; 86.6)
	…	…	…	PvEV-2; dsRNA; Alphaendornavirus (AB719398; 97.5)
	…	…	…	CPMMV; +ssRNA; Carlavirus (KC774020; 68.4)
	…	…	…	CMV; +ssRNA; Cucumovirus (KJ400004; 86.0)
	…	…	…	CMoV; 1ssRNA; Umbravirus (CED51824; 51.5)
	…	…	…	CMoMV; 1ssRNA; Umbravirus (ACJ03575; 56.1)
	…	…	…	OPMV; 1ssRNA; Umbravirus (AHZ65104; 33.0)
	…	…	…	ETBTV; 1ssRNA; Umbravirus (AIL27641; 33.1)
	…	…	…	TBTV; 1ssRNA; Umbravirus (TBTV; 77.9)
NZ	HXH-3	5,286,206	203,035	BCMNV; +ssRNA; Potyvirus (AY864314; 86.1)
	…	…	…	BCMV; +ssRNA; Potyvirus (KF114860; 99.9)
	…	…	…	BnYDV; +ssRNA; Crinivirus (EU191905; 77.9)
	…	…	…	CPMMV; +ssRNA; Carlavirus (KJ534277; 73.6)
	…	…	…	NCMV; –ssRNA; Cytorhabdovirus (ADE61669; 24.6)
LZ	HXH-6	11,658,110	378,180	BCMNV; +ssRNA; Potyvirus (AY864314; 95.8)
	…	…	…	CABMV; +ssRNA; Potyvirus (DQ397527; 89.2)
	…	…	…	ToLCArV; +/–ssDNA; Begomovirus (DQ519575; 24)
	…	…	…	ToLCYTV; +/–ssDNA, (AJ865340; 19.3)
	…	…	…	ToLCUV; +/–ssDNA; Begomovirus (DQ127170; 62.8)
	…	…	…	PvEV-1; dsRNA; Alphaendornavirus (KT456287; 61.8)
	…	…	…	PvEV-2; dsRNA; Alphaendornavirus (AB719398; 94.2)
	HXH-7	7,989,740	80,846	TMoV; +ssRNA; Umbravirus (AY007231; 91.1)
	…	…	…	PvEV-1; dsRNA; Alphaendornavirus (KT456287; 91.0)
	…	…	…	PvEV-2; dsRNA; Alphaendornavirus (AB719398; 94.7)
	…	…	…	RuFDV; dsDNA-RT; unassigned (ACL36982; 23.1)
	…	…	…	HRLV; dsDNA-RT; Caulimovirus (AAW56089; 14.3)
	…	…	…	CERV; dsDNA-RT; Caulimovirus (ABX80503; 23.2)
	…	…	…	EVCV; dsDNA-RT; unassigned (ACB69773; 13.1)
	…	…	…	MMV; dsDNA-RT; Caulimovirus (AAM53128; 22.6)
	…	…	…	DMV; dsDNA-RT; Caulimovirus (ABW80581; 24.1)
	…	…	…	SbCMV; dsDNA-RT; Soymovirus (CAA33833; 14.2)
	…	…	…	PEMV; 1ssRNA; Umbravirus (AAU20330; 22.3)
	…	…	…	SVBV; dsDNA-RT; Caulimovirus (AKB94072; 36.7)
	…	…	…	GRV; 1ssRNA; Umbravirus (CTQ57207; 33.7)
	…	…	…	SPuV; dsDNA-RT; Caulimovirus (AFP95350; 15.9)
WZ	HXH-15	28,223,699	3,793,494	BCMNV; +ssRNA; Potyvirus (AY864314; 77.2)
	…	…	…	SBMV; +ssRNA; Sobemovirus (DQ875594; 98.5)
	…	…	…	PvEV-1; dsRNA; Endornavirus (KT456287; 99.3)

a SHZ, EZ, NZ, LZ, and WZ indicate agricultural research zones in Tanzania: southern highlands, eastern, northern, Lake, and western zones, respectively.

b Database searches for viruses in bold type was achieved using the Blastx approach. Abbreviations: ss = single-stranded and ds = double-stranded RNA or DNA, BCMNV = Bean common mosaic necrosis virus, BCMV = Bean common mosaic virus, PeMoV = Peanut mottle virus, SBMV = Southern bean mosaic virus, PvEV-1 = Phaseolus vulgaris alphaendornavirus 1, PvEV-2 = Phaseolus vulgaris alphaendornavirus 2, CPMMV = Cowpea mild mottle virus, CMV = Cucumber mosaic virus, CMoV = Carrot mottle virus, CMoMV = Carrot mottle mimic virus, OPMV = Opium poppy mosaic virus, ETBTV = Ethiopian tobacco bushy top virus, TBTV = Tobacco bushy top virus, CABMV = Cowpea aphid-borne mosaic virus, BnYDV = Bean yellow disorder virus, NCMV = Northern cereal mosaic virus, ToLCArV = Tomato leaf curl Arusha virus, ToLCYTV = Tomato leaf curl Mayotte virus, ToLCUV = Tomato leaf curl Uganda virus, TMoV = Tobacco mottle virus, RuFDV = Rudbeckia flower distortion virus, HRLV = Horseradish latent virus, CERV = Carnation etched ring virus, EVCV = Eupatorium vein clearing virus, MMV = Mirabilis mosaic virus, DMV = Dahlia mosaic virus, SbCMV = Soybean chlorotic mottle virus, PEMV = Pea enation mosaic virus, GRV = Groundnut rosette virus, SVBV = Strawberry vein banding virus, and SPuV = Soybean putnam virus. For sample HXH-2, viruses CMoV, CMoMV, OPMV, ETBTV, and TBTV (all belonging to genus Umbravirus) identified through Blastx are most likely sequences of one and the same novel virus.

Blastn and Blastx revealed viruses belonging to 11 genera: Poty-virus (Potyviridae), Sobemovirus (unassigned), Alphaendornavirus (Endornaviridae), Carlavirus (Betaflexiviridae), Cucumovirus (Bromoviridae), Umbravirus (Tombusviridae), Crinivirus (Closteroviridae), Begomovirus (Geminiviridae), Cytorhabdovirus (Rhabdoviridae), and Caulimovirus and Soymovirus (Caulimoviridae) (Supplementary Fig. S2). The Blast searches using contig sequences obtained in this study matched sequences of over 32 viruses in the database. The coverage, sequencing depth, and number of contigs for these viruses were different (Supplementary Table S2).

The sequencing depths ranged from 5.2x for Bean yellow disorder virus (BnYDV; Crinivirus) in sample HXH-3 to 5,632x for SBMV in sample HXH-15. Some contig sequences obtained through de novo assembly were related to more than one virus in the sequence database (e.g., an umbravirus in sample HXH-2); the most likely viruses infecting plants whose RNA was included in the sequenced samples are shown in Table 3. Therefore, the NGS contigs revealed that common bean plants in Tanzania were infected by 15 viruses (Table 3).

The commonest viruses in samples were BCMNV, PvEV-1, and PvEV-2, which were detected in samples from at least three agricultural research zones (Table 3). BCMNV was detected in samples from all five agricultural research zones. PvEV-1 and PvEV-2 were detected in pooled RNA samples HXH-1 (southern highlands zone), HXH-2 (eastern zone), and HXH-6 and HXH-7 (Lake zone). PvEV-1 but not PvEV-2 was detected in the pooled RNA sample from the western zone (HXH-15). Neither PvEV-1 nor PvEV-2 was detected in sample HXH-3 (northern zone). BCMV, widely reported to infect common bean worldwide, was detected in samples from the eastern (HXH-2) and northern (HXH-3) zones but not from the southern highlands (HXH-1), Lake (HXH-6 and HXH-7), and western zones.

Other viruses detected were CPMMV (HXH-2 and HXH-3), PeMoV (HXH-1, accession numbers MF784805 and MF784806), SBMV (HXH-1, accession numbers MF784807 and MF784807; and HXH-15, accession number MG344643), CABMV (HXH-6), BnYDV (HXH-3), CMV (HXH-2), unidentified umbravirus closely related to Carrot mottle mimic virus (CMoMV), Carrot mottle virus (CMoV), Opium poppy mosaic virus (OPMV), Ethiopian tobacco bushy top virus (ETBTV), Tobacco bushy top virus (TBTV) (HXH-2), Tobacco mottle virus (TMoV) (HXH-7), Northern cereal mosaic virus (NCMV) (HXH-3), and a caulimovirus most closely related to Strawberry vein banding virus (SVBV) and other caulimo-viruses (HXH-7) (Table 3). A sequence with some similarity to bego-moviruses (Tomato leaf curl Uganda virus [ToLCUV]) was found in sample HXH-6. Further PCR and Sanger sequencing work confirmed that a ToLCUV-related begomovirus was found in only one common bean sample.

NGS sequences of BCMNV. De novo assembly of NGS data generated complete or nearly complete genomes for BCMNV. In the pooled RNA sample HXH-1, a contig of 9.6 kb (insert size 21; accession number MF078483) was obtained. Alignment against the complete sequence of strain TN1 (accession number KY659306) showed it to be a complete sequence and that the two sequences were 99% similar at both nucleotide and amino acid sequence levels. In the pooled RNA sample HXH-2, the contig obtained from reads of size nt (9.2 kb; accession number MF405187) was closely related to a published sequence of BCMNV isolate 1755b (accession number KY659305). This contig sequence translated to yield a partial first potyviral protein (P1) and the other nine typical potyviral proteins (Adams et al. 2005). In the samples collected from Tarime (pooled RNA sample HXH-6), a 9.4-kb sequence (accession number MF405189) was obtained. It was closely related (99% similar at nucleotide level) to isolate 1755b (Feng et al. 2017; accession number KY659305; pathogenic group VI) and also strain TN1 (accession number HQ229995; 99% similarity at nucleotide level). In a nonpooled RNA sample (HXH-5), a 9.3-kb sequence (accession number MF405192) was obtained and found to be closely related to published isolate 1755b (accession number KY659305). Thus, in all common bean growing areas in Tanzania, the NGS-based sequences of BCMNV isolates were closely related to the sequences of a strain in pathogenic group VI (Feng et al. 2017). Comparison of these four sequences revealed that they were 98.3 to 99.0 and 99.2 to 99.7% identical at nucleotide (9,248 nt, including 3' untranslated region) and amino acid (3,001 amino acid) levels, respectively, suggesting low sequence variability within BCMNV isolates in Tanzania.

NGS sequences of BCMV. De novo assembly of NGS data generated a sequence of 10,051 nt (excluding the poly-A tail) for pooled RNA sample HXH-2 (eastern zone). This sequence (combined inserts; accession number MF405191) was 99% similar to that of strain NL-1 (accession number AY112735). In another pooled RNA sample, HXH-3, a 7.1-kb contig was obtained (accession number MF405188) and was closely related to sequence of strain NL-1 (accession number AY112735).

In a nonpooled RNA sample (HXH-4), a complete sequence of BCMV (10,003 nt; isolate TZ:Mor533:2015; accession number MF405190) was obtained by aligning contigs obtained by de novo assembly of read size 21 nt and combined inserts using SeqMan v. 5.03. National Center for Biotechnology Information Blast showed it was 92% similar to isolate RU1 (accession number GQ219793) (Naderpour et al. 2009), which is a revised sequence of a recombinant isolate originally sequenced by Larsen et al. (2005) (accession number AY864314). Because this NGS-based sequence was obtained by assembling several contigs, we designed three pairs of primers to target the potyviral P3, CI, and CP genomic regions, and RT-PCR and sequencing confirmed that the NGS sequence was identical in corresponding regions of three Sanger-based sequences of this isolate: MF784802, MF784803, and MF784804. These Sanger sequences were <95.5% similar to potyviral sequences in GenBank. One common bean plant infected with TZ:Mor533:2015 isolate had severe necrotic symptoms on leaves.

**Fig. 3:**
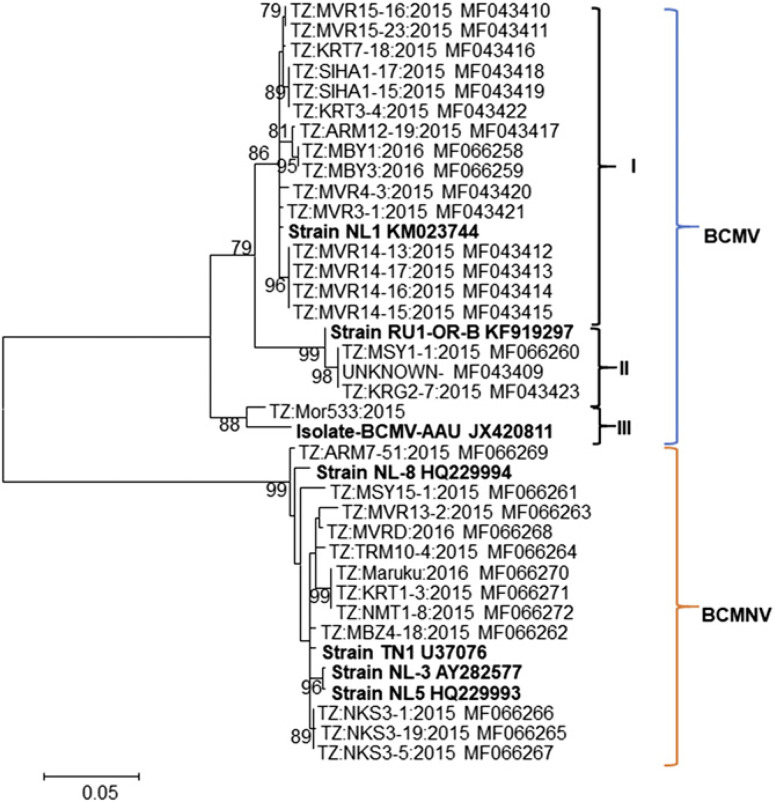
Phylogenetic tree generated using the coat protein nucleotide sequences (620 nucleotides) of *Bean common mosaic virus* (BCMV) and Bean common mosaic necrosis virus (BCMNV) isolates. The tree is drawn to scale, with branch lengths measured in the number of substitutions per site. Numbers at branches represent bootstrap values of 1,000 replicates, of which only values of >60% are shown. Isolate names are shown along with the accession numbers assigned to their sequences in this study (nonbold text) and in previous studies (bold text).

CP sequence variability of BCMNV and BCMV. Sanger sequencing was used to generate 31 partial CP-encoding sequences of BCMNV (n =12) and BCMV (n = 19). These sequences were used to generate a phylogenetic tree for BCMNV and BCMV isolates. Phylogenetic analysis of these sequences (620 nt in size) at the conserved 3' end resulted in two groups of virus species: BCMV and BCMNV (Fig. 3). Furthermore, the BCMV group consisted of three subclusters. Considering only Tanzanian isolates, one subcluster consisted of 15 isolates and the other two consisted of 3 isolates (TZ:KRG2-7:2015, TZ:MSY1-1:2015, and unknown) and a single isolate (TZ:Mor533:2015) from Tanzania. The isolates of strain NL-1 were predominant. Isolates TZ:KRG2-7:2015 and TZ: MSY1-1:2015, which clustered with the Russian strain (accession number KF919297) (Feng et al. 2014), were collected from the Lake zone. The nucleotide sequences (782 nt) of the 19 BCMV isolates were 90.2 to 100% similar to each other (Table 4). The same sequences were 91.9 to 100% similar at the amino acid level. The CP sequence of isolate TZ:Mor533:2015 was 90.2 to 91.1% similar to sequences of all other isolates. For the amino acid sequences, this isolate was closely related to the RU1-like isolate TZ:KRG2-7:2015 collected from the Lake zone. The analysis of CP-encoding sequences of BCMNV (626 nt; starting at position 78, with reference to the sequence of isolate TZ:MSY15-1:2015; accession number MF066261) showed genetic variation ranging within 97.1 to 100 and 99.0 to 100% at nucleotide and amino acid levels, respectively (Table 5).

**Table 4:** Nucleotide and amino acid sequence similarities among Bean common mosaic virus (BCMV) isolates^a^

Seq	A	B	C	D	E	F	G	H	I	J	K	L	M	N	O	P	Q	R	S
A	…	100	91.5	91.0	98.3	98.3	97.8	97.8	97.8	97.6	98.2	98.8	98.3	98.3	98.0	98.2	98.3	91.0	90.2
B	100	**	91.5	91.0	98.3	98.3	97.8	97.8	97.8	97.6	98.2	98.8	98.3	98.3	98.0	98.2	98.3	91.0	90.2
C	93.4	93.4	**	99.3	92.0	92.0	91.8	91.8	91.8	91.6	91.9	92.0	91.9	91.9	92.0	92.1	91.9	99.3	90.2
D	92.6	92.6	98.8	**	91.5	91.5	91.3	91.3	91.3	91.1	91.4	91.5	91.4	91.4	91.5	91.6	91.4	100	90.5
E	98.8	98.8	93.4	92.6	**	100	98.9	98.9	98.9	98.8	99.6	99.2	99.6	99.6	99.2	99.3	99.6	91.5	91.1
F	98.8	98.8	93.4	92.6	100	**	98.9	98.9	98.9	98.8	99.6	99.2	99.6	99.6	99.2	99.3	99.6	91.5	91.1
G	98.4	98.4	93.0	92.3	98.8	98.8	**	100	100	99.8	98.8	98.7	98.8	98.8	98.9	99.3	98.8	91.3	90.6
H	98.4	98.4	93.0	92.3	98.8	98.8	100	**	100	99.8	98.8	98.7	98.8	98.8	98.9	99.3	98.8	91.3	90.6
I	98.4	98.4	93.0	92.3	98.8	98.8	100	100	**	99.8	98.8	98.7	98.8	98.8	98.9	99.3	98.8	91.3	90.6
J	98.0	98.0	92.6	91.9	98.4	98.4	99.6	99.6	99.6	**	98.7	98.5	98.7	98.7	98.8	99.2	98.7	91.1	90.5
K	98.8	98.8	93.4	92.6	100	100	98.8	98.8	98.8	98.4	**	98.8	99.4	99.4	99.1	99.2	99.4	91.4	91.0
L	99.2	99.2	93.8	93.0	99.6	99.6	99.2	99.2	99.2	98.8	99.6	**	98.9	98.9	98.9	99.1	98.9	91.5	90.9
M	98.8	98.8	93.4	92.6	100	100	98.8	98.8	98.8	98.4	100	99.6	**	100	99.1	99.2	100	91.4	90.7
N	98.8	98.8	93.4	92.6	100	100	98.8	98.8	98.8	98.4	100	99.6	100	**	99.1	99.2	100	91.4	90.7
O	99.2	99.2	93.8	93.0	99.6	99.6	99.2	99.2	99.2	98.8	99.6	100	99.6	99.6	**	99.3	99.1	91.5	90.9
P	98.8	98.8	93.4	92.6	99.2	99.2	99.6	99.6	99.6	99.2	99.2	99.6	99.2	99.2	99.6	**	99.2	91.6	90.7
Q	98.8	98.8	93.4	92.6	100	100	98.8	98.8	98.8	98.4	100	99.6	100	100	99.6	99.2	**	91.4	90.7
R	92.6	92.6	98.8	100	92.6	92.6	92.3	92.3	92.3	91.9	92.6	93.0	92.6	92.6	93.0	92.6	92.6	**	90.5
S	92.3	92.3	94.2	95.3	92.6	92.6	92.3	92.3	92.3	91.9	92.6	93.0	92.6	92.6	93.0	92.6	92.6	95.3	**

a Genetic variability among BCMV isolates. Percent nucleotide (upper triangle) and amino acid (lower triangle) sequence similarities among Tanzanian isolates. The BCMV coat protein nucleotide sequence length used was 782 nucleotides (starting with the 52nd nucleotide with reference to isolate TZ:KRG2-7:2015). The corresponding amino acids were used to determine amino acid sequence identity. Letters A to S represent, in order, the following isolates: TZ:MBY1:2016 (MF066258), TZ:MBY3:2016 (MF066259), TZ:MSY1-1:2015 (MF066260), UNKNOWN (MF043409), TZ:MVR15-16:2015 (MF043410), TZ:MVR15-23: 2015 (MF043411), TZ:MVR14-13:2015 (MF043412), TZ:MVR14-17:2015 (MF043413), TZ:MVR14-16:2015 (MF043414), TZ:MVR14-15:2015 (MF043415), TZ:KRT7-18:2015 (MF043416), TZ:ARM1219:2015 (MF043417), TZ:SIHA117:2015 (MF043418), TZ:SIHA115:2015 (MF043419), TZ: MVR4-3:2015 (MF043420), TZ:MVR3-1:2015 (MF043421), TZ:KRT3-4:2015 (MF043422), TZ:KRG2-7:2015 (MF043423), and TZ:Mor533:2015 (MF784804).

**Table 5:** Nucleotide and amino acid sequence similarities among Bean common mosaic necrosis virus (BCMNV) isolates^a^

Seq	A	B	C	D	E	F	G	H	I	J	K	L	M
A	**	97.9	97.7	97.4	97.9	98.0	98.0	97.9	97.9	97.1	97.1	97.1	97.6
B	99.5	**	98.2	98.8	99.3	99.5	99.5	99.0	98.4	98.5	98.5	98.5	99.0
C	99.5	100	**	98.7	98.2	98.4	98.4	98.8	97.6	98.0	98.0	98.0	97.9
D	99.0	99.5	99.5	**	98.8	99.0	99.0	99.2	98.2	98.7	98.7	98.7	98.5
E	99.0	99.5	99.5	99.0	**	99.8	99.8	99.0	98.4	98.5	98.5	98.5	99.0
F	99.5	100	100	99.5	99.5	**	100	99.2	98.5	98.7	98.7	98.7	99.2
G	99.5	100	100	99.5	99.5	100	**	99.2	98.5	98.7	98.7	98.7	99.2
H	99.5	100	100	99.5	99.5	100	100	**	98.4	98.8	98.8	98.8	98.7
I	99.5	100	100	99.5	99.5	100	100	100	**	97.6	97.6	97.6	98.0
J	99.5	100	100	99.5	99.5	100	100	100	100	**	100	100	98.2
K	99.5	100	100	99.5	99.5	100	100	100	100	100	**	100	98.2
L	99.5	100	100	99.5	99.5	100	100	100	100	100	100	**	98.2
M	99.0	99.5	99.5	99.0	99.0	99.5	99.5	99.5	99.5	99.5	99.5	99.5	**

a Genetic variability among BCMNV isolates. Percent nucleotide (upper triangle) and amino acid (lower triangle) sequence similarities among Tanzanian isolates. The BCMNV coat protein nucleotide sequence length used was 626 nucleotides. Letters A to M represent the following isolates in order: TZ:MSY15-1:2015 (MF066261), TZ:MBZ4-18:2015 (MF066262), TZ:MVR13-2:2015 (MF066263), TZ:TRM10-4:201 (MF066264), TZ:NKS3-19:2015 (MF066265), TZ: NKS3-1:2015_MF066266, TZ:NKS3-5:2015 (MF066267), TZ:MVRD:2016 (MF066268), TZ:ARM7-51:2015 (MF066269), TZ:Maruku:2016 (MF066270), TZ: KRT1-3:2015 (MF066271), TZ:NMT1-8:201 (MF066272), and strain TN1 (HQ229995).

## Discussion

We detected 15 viruses belonging to 11 genera in the common bean samples collected from all over Tanzania. They included viruses reported in previous surveys of common bean virus diseases in the country—BCMNV, BCMV, CMV, and CPMMV (Mink and Keswani 1987; Njau and Lyimo 2000; Njau et al. 2006; Vetten and Allen 1991)—and the most recently reported seedborne, cryptic viruses, PvEV-1 and PvEV-2 (Nordenstedt et al. 2017). Other viruses detected have been reported to infect plants other than common bean in Tanzania: CABMV (Potyvirus) in cowpea (Vigna unguiculata (L.) Walp) (Patel and Kuwite 1982) and CMV in cucurbits (Sydanmetsa and Mbanzibwa 2016). There are no documented occurrences in Tanzania of some detected viruses, including SBMV, umbraviruses (ToMV and CMoMV), and ToLCUV-related begomovirus. Bego-moviruses have been reported to infect common bean in Latin America, and viruses that infect tomato can also infect common bean (Macedo et al. 2017), but not in Africa. Previously, the use of ELISA had limited our knowledge of viruses infecting common bean in Tanzania and did not generate molecular evidence to confirm occurrence of specific viruses (Mwaipopo et al. 2017). The use of NGS not only helped reveal viruses infecting common bean in Tanzania but also generated much-needed molecular information for development of diagnostic tools for virus disease management. Indeed, primers developed and used in this work are being used in other studies.

Discovery of viruses through de novo assembly of NGS data requires that a database contains previously submitted sequences that are related to the query contig sequences. In this study, some detected viruses could only be identified to the genus level because of low identities. In some instances, a certain contig matched more than one virus sequence in the database. When a contig matches more than one virus in a database with low coverages and similarities, it indicates that it is only related to those viruses and that it could be a sequence of a different virus or strain. Therefore, some identified viruses may not be exactly the same as those infecting common bean in Tanzania but, instead, closely or distantly related viruses or strains, for which sequences have not been deposited in sequence databases. Examples from our study include an umbravirus (pooled RNA sample HXH-2) and a caulimovirus (pooled RNA sample HXH-7). Moreover, it is worth noting that small RNA were extracted from samples stored in silica gel or dried using a plant press. We are unaware of previous work that used samples stored under similar conditions but it is reasonable that the use of dry leaf samples could affect the amount of small RNA extracted and, thus, the coverage and viruses detected. However, in this study, it was possible to obtain complete or nearly complete nucleotide sequences from some of these samples.

NGS technology is still in its infancy, and the possibility of sequencing errors and further problems is expected during de novo assembly of reads. There are concerns that sequences derived from pools of plant material can be chimeras artificially assembled from pieces of multiple viruses (Roossinck et al. 2015; Simmonds et al. 2017). We cannot discount this possibility; however, some sequences obtained in this study were over 99% identical to Sanger sequences in nucleotide databases. Moreover, for one isolate (TZ:Mor533:2015), the three randomly selected genomic regions that were Sanger sequenced were identical to the NGS-based sequence. Additionally, primers designed using NGS sequences worked perfectly for specific detection of viruses (e.g., CPMMV, SBMV, and ToLCUV-related begomovirus).

De novo assembly for detection of plant viruses is normally done on reads of 21 to 24 nt contained in one fastq file. In the present study, combined and separate read sizes (21 to 24 nt) were analyzed. The viruses detected following analysis of combined inserts or separate reads of sizes 21 to 24 nt were the same (especially for sizes 21 and 22 nt) but the contigs obtained differed in size. Interestingly, when the contigs overlapped, they were identical to each other. Although not a subject of this work, it is noteworthy that SBMV was still reliably detectable in reads of >25 nt but not >35 nt. Analyzing sequences first as combined reads and then separately enabled the obtaining of two sequences for alignment and manual editing. Furthermore, it took less time to assemble sequences and less computational capability was required when reads of different sizes were analyzed separately. This is particularly important in developing countries where supercomputers are generally lacking (Mwaipopo et al. 2017).

Previously, BCMV and BCMNV were the most commonly reported viruses of common bean in Tanzania (Myers et al. 2000; Njau and Lyimo, 2000). Thus, it was reasonable to focus on determining their distribution in the country and incidence in common bean fields. Whereas visual assessment indicated that the incidence of virus diseases could be as high as 98%, RT-PCR-based assessment revealed the incidence of diseases caused by BCMNV and BCMV in the range of 0.0 to 76.7 and 0.0 to 36.7%, respectively. These viruses were more abundant in samples collected from the eastern and northern zones than from the Lake, western, and southern highlands zones. The districts Wanging’ ombe, Mbinga, and Namtumbo in the southern highlands were nearly free of BCMNV and BCMV. Myers et al. (2000) found low incidence of BCMNV in southern Tanzania, which they attributed to low vector population densities due to the high altitudes of these areas. Data obtained in this study suggested that mixed infections between BCMNV and BCMV were rare in Tanzania, in agreement with previous observations; however, coinfections can be high elsewhere (Vetten and Allen 1991). In Mexico, Chiquito-Almanza et al. (2017) found a 7% mixed infection in common bean samples collected from fields. In all agricultural research zones in the current study, visual assessment suggested that virus diseases were commonplace in common bean fields.

The observed differences in visually assessed and RT-PCR-based incidence levels can be explained by occurrence of pathogenic viruses other than BCMNV and BCMNV, as revealed by NGS data. Findings of visual symptom incidence higher than virus incidence in samples were also reported by Segundo et al. (2008), who did not detect any viruses in 61% of samples from plants with virus-like disease symptoms. There are different possible reasons for this discrepancy. Viruses such as CPMMV and SBMV, for example, cause mild to severe disease symptoms on common bean genotypes in Tanzania (our unpublished results) and could be responsible for some of the observed symptoms. Moreover, some symptoms on plants caused by edaphic factors (e.g., nutrient toxicities) can be confused with virus disease symptoms (Kennelly et al. 2012).

Incidence of BCMV and BCMNV was relatively low in the western (Kigoma) and southern highlands areas. The factors driving the spread of viruses that infect common bean in Tanzania remain largely unknown. Some viruses detected in this work are known to be transmitted through seed at different efficiencies (Njau and Lyimo 2000; Okada et al. 2013). Njau and Lyimo (2000) reported incidence of 12.4 and 36.6% for BCMNV and BCMV, respectively, in seed lots collected from farmers and research centers in Tanzania. However, a recent study showed that pathogenic viruses—but not CPMMV—are rarely transmitted in seed of different common bean varieties collected from farmers in Tanzania (Nordenstedt et al. 2017). This rare transmission of pathogenic viruses was associated with efforts invested in breeding for resistance (Kusolwa et al. 2016). It is understood that the rate of virus transmission in seed is not always a good indicator of disease epidemiology because, in the presence of vectors, low seed transmission is sufficient to spread viruses and cause disease epidemics (Johansen et al. 1994). The observed variation in virus incidence could be attributed to the wide range of cultivars grown in Tanzania (Fivawo and Msolla 2011; Nordenstedt et al. 2017). However, for a variety of reasons that include lack of awareness and cost avoidance, some farmers are reluctant to adopt and plant new varieties or use certified and Quality Declared Seeds. Other factors likely to affect levels of virus disease incidence in the area are the availability of alternative hosts and vectors for viruses (Spence and Walkey 1995). These play an important role in new virus infections in different cropping seasons. For example, in Tanzania, BCMNV, BCMV, CMV, and CABMV have been detected in hosts other than common bean (Myers et al. 2000; Patel and Kuwite 1982). Very low incidence of BCMNV and absence of BCMV in the western zone could be partly due to the isolation of the area from other common bean growing areas in Tanzania. The interregional (within-country) bean seed trade may not lead to virus spread because seedborne virus diseases are rare (Nordenstedt et al. 2017). Considering all of this information, it was not surprising to find differences in virus disease incidence between two fields located in close proximity and within or between agricultural research zones.

The RT-PCR results showed that BCMV was present in all zones, except the western zone. However, NGS data indicated absence of BCMV in the southern highlands, western, and Lake zones. This discrepancy can be explained by low incidence of BCMV in common bean plants in Tanzania as well as a small sample size (30 plants per zone) used in NGS; it is not related to the detection sensitivity of NGS and RT-PCR methods. Therefore, it is likely that the chance of detection of BCMV, and possibly other viruses, may rise with an increased number of pooled RNA samples per zone.

The results showed that isolates of BCMV were more diverse than those of BCMNV. Occurrence of many strains of BCMV has been known for quite a long time through studies on genetic interaction between this virus and common bean genotypes (Drijfhout 1978). Using differential cultivars, it has been shown that the strains NL1, NL3, NL5, NL8, TN1, TN2, and TN3 occur in Tanzania (Njau and Lyimo 2000; Spence and Walkey 1994; Vetten and Allen 1991). In our study, RT-PCR revealed the isolates of strain NL1 could be the most dominant isolates in Tanzania. The other strain that was detected was RU1. Isolate TZ:Mor533:2015 appears to be genetically distinct from all other sequenced isolates but, at the amino acid sequence level, it was closely related to the RU1-like isolate TZ: KRG2-7:2015. This may suggest occurrence of distinct strains of RU1 isolates in Tanzania. We did not analyze for recombination given the fact that sequences were obtained through NGS but the BCMV isolate TZ:Mor533:2015 was related to recombinant isolates. Occurrence of natural recombinant isolates of BCMV is common and some recombinant isolates can overcome resistance conferred by such genes as bc-2 and bc-3 (Feng et al. 2014, 2015). High genetic variability among BCMV isolates means that common bean genotypes bred for resistance to this virus must be challenged against a wide range of isolates before being considered for release.

During field surveys, we observed that common bean plants in Tanzania exhibit severe virus disease symptoms of unknown cause. Laboratory analysis revealed that, apart from BCMV and BCMNV, there were other pathogenic viruses (e.g., CPMMV, PeMoV, CABMV, SBMV, and ToLCUV) infecting common bean plants that could be responsible for the unexplained symptoms in fields throughout the country. Thus, plant breeders and pathologists should investigate the damage and yield losses caused by these viruses and also consider any possible interactions between pathogenic viruses and viruses whose pathogenic roles remain undetermined (e.g., PvEV-1 and PvEV-2). We anticipate that the results reported herein will form the basis for future studies on common bean viruses as well as guide plant breeders and plant pathologists in developing strategies for management of common bean virus diseases in Tanzania. For example, this information can be used in selecting seed multiplication sites or deploying planting material. To the best of our knowledge, this work represents the first comprehensive survey for common bean viruses in Tanzania and provides unprecedented molecular evidence for occurrence of different viruses in common bean plants in the country.
